# Cognitive emotion regulation: characteristics and effect on quality of life in women with breast cancer

**DOI:** 10.1186/s12955-015-0242-4

**Published:** 2015-05-06

**Authors:** Lingyan Li, Xiongzhao Zhu, Yanjie Yang, Jincai He, Jinyao Yi, Yuping Wang, Jinqiang Zhang

**Affiliations:** Medical Psychological Institute, Second Xiangya Hospital, Central South University, Changsha, Hunan 410011 P.R. China; National Technology Institute of Psychiatry, Central South University, Changsha, China; Department of Medical Psychology, Public Health Institute of Harbin Medical University, Harbin, China; The First Affiliated Hospital, Wenzhou Medical College, Wenzhou, China

**Keywords:** Cognitive coping, Emotion regulation, Quality of life, Breast cancer, Oncology, Women’s health

## Abstract

**Background:**

In recent decades, researchers and clinicians have sought to determine how to improve the quality of life (QOL) of women with breast cancer. Previous research has shown that many women have particular behavioral coping styles, which are important determinants of QOL. As behavior is closely associated with cognition, these patients may also have particular cognitive coping styles. However, the cognitive coping characteristics and their effects on QOL in women with breast cancer remain unclear. Thus, this study aimed to characterize cognitive coping styles among women with breast cancer and explore the effects of cognitive emotion regulation strategies on QOL.

**Methods:**

The Chinese version of the Cognitive Emotion Regulation Questionnaire was used to assess cognitive coping strategies in 665 women newly diagnosed with breast cancer and 662 healthy women. QOL of patients was assessed using the Functional Assessment of Cancer Therapy for Breast Cancer Scale. Independent-samples *t*-tests were performed to investigate group differences in reporting of cognitive coping strategies. Multiple regression analyses were performed to examine the effects of cognitive coping strategies on QOL in patients after controlling for sociodemographic and medical variables.

**Results:**

Compared with control subjects, patients reported less frequent use of self-blame, rumination, positive refocusing, refocusing on planning, positive reappraisal, and blaming others, and more frequent use of acceptance and catastrophizing (all *p* < 0.01). The three strongest predictors of group membership were catastrophizing (B = −0.35), acceptance (B = −0.29), and positive reappraisal (B = 0.23). All nine coping strategies were significantly correlated with QOL in patients (all *p* < 0.05). After controlling for sociodemographic and medical variables, self-blame, rumination, and catastrophizing negatively affected QOL (all *p* < 0.05), whereas acceptance and positive reappraisal had positive effects (all *p* < 0.01).

**Conclusions:**

Compared with healthy women, women newly diagnosed with breast cancer use catastrophizing and acceptance more frequently, and positive reappraisal, self-blame, rumination, positive refocusing, refocusing on planning, and blaming others less frequently. Catastrophizing, rumination, and self-blame may be not conducive to QOL of women with breast cancer and acceptance and positive reappraisal may be useful.

## Background

Breast cancer is the most commonly diagnosed cancer in women worldwide [1]. With improvements in detection and treatment, the survival rate of breast cancer has increased dramatically since the 1990s [2], but patients’ quality of life (QOL) continues to be affected by stressors induced, for example, by exposure to treatment side effects [3,4]. Survival is not sufficient; patients also want to live well. QOL has also been shown to be a significant prognostic factor for mortality and cancer recurrence [5]. Thus, researchers have faced the important problem of how to improve the QOL of women with breast cancer in recent decades.

QOL is a multidimensional concept involving aspects of individuals’ physical, psychological, and social well-being [6]. The determinants of QOL in women with breast cancer include psychosocial factors, such as coping style, as well as sociodemographic and medical factors [7]. Interventions are presumed to be capable of changing psychosocial factors and thereby QOL outcomes [8,9], whereas achieving change in the other two factor categories is generally difficult.

According to psychological stress theories [10], coping is the main mediator between stressful events and outcomes. Coping is defined as “an individual’s efforts (both behavioral and cognitive) to manage demands (condition of harm, threat or challenge) that are appraised (or perceived) as exceeding or taxing his or her resources” [10]. Garnefski et al. [11] argued that all coping efforts can be classified broadly as emotion regulation, which refers to a wide range of biological, social, behavioral, and conscious and unconscious cognitive processes. Previous studies, focused primarily on behavioral coping, have shown that different coping strategies had distinct effects on QOL in women with breast cancer [12,13], but they did not recognize the importance of the cognitive component of the coping process. Garnefski et al. [11] reported that this cognitive component may help patients manage or regulate emotions or feelings to avoid becoming overwhelmed, and they defined cognitive emotion regulation strategies as the conscious mental strategies that individuals use to cope with the intake of emotionally arousing information. In some situations, cognitive coping is more important than other coping strategies; for example, Kraaij et al. [14] found that cognitive coping strategies had stronger effects than behavioral coping strategies on emotional problems in patients with definitive infertility, and they suggested that intervention programs should place more emphasis on cognitive techniques.

Certain cognitive emotion regulation strategies that an individual uses to deal with a life stressor may be associated with psychological distress and QOL [15,16]. For example, Garnefski et al. [16] found that rumination was associated not only with the reporting of internalizing problems, but also with lower health-related QOL. Some studies have found that cognitive emotion regulation strategies accounted for considerable variance in psychological adjustment and somatic symptoms in women with breast cancer; the strategies of acceptance, positive refocusing, and positive reappraisal may be beneficial, whereas the strategy of catastrophizing may not be useful [13,17-20].

A previous study found that the behavioral coping style most often observed among women with breast cancer was extreme suppression of feelings, which negatively affected prognosis [21]. As individuals’ behaviors are closely associated with cognition, women with breast cancer may also have a particular cognitive coping style, which may have important effects on QOL. However, the characteristics of cognitive coping styles in these women remain unclear. To our knowledge, no study has investigated how cognitive emotion regulation styles among women with breast cancer relate to QOL. Thus, the aims of this study were to characterize cognitive emotion regulation styles among women with breast cancer and to explore the effects of cognitive emotion regulation strategies on QOL. As previous studies have found that people with physical and mental diseases report more rumination and catastrophizing and less positive reappraisal than do healthy control subjects [22,23], we hypothesized that women with breast cancer would exhibit this pattern of cognitive coping. As the core of behavioral coping among women with breast cancer is the acceptance and control of emotions [21], we hypothesized that these patients would use cognitive coping strategies such as acceptance more than they use the strategy of blaming others. According to previous findings regarding psychological adjustment and somatic symptoms in women with breast cancer [17-20], we hypothesized that rumination and catastrophizing would negatively affect QOL, whereas positive reappraisal, positive refocusing, and acceptance would be beneficial to QOL.

## Methods

The study was conducted from January 2011 to June 2012 and approved by the Ethics Committee of the Second Xiangya Hospital, Central South University.

### Participants

#### Patient sample

Eligible women who had been undergoing treatment for breast cancer at two hospitals in Changsha, Hunan Province, China, were invited to participate in this study. Eligible patients met the following criteria: (1) diagnosed with and informed of stage I or II breast cancer within a month (by biopsy), (2) receipt of treatment, and (3) ability to speak Chinese. Patients with the following conditions were excluded: (1) breast cancer recurrence, (2) known untreated or unstable major medical condition other than breast cancer, (3) known major psychiatric or neurological disorder that would interfere with completion of the measures, and (4) history of substance abuse.

**Figure 1:**
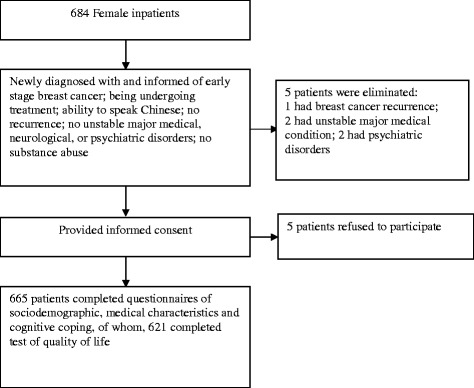
**Process for patient selection.**

The final sample included 665/684 (97.2%) patients who were invited to participate in the study; five patients declined participation after being informed of the study aims and procedure, five patients met one or more exclusion criteria, and nine patients did not complete the questionnaires (see Figure 1). Participant age ranged from 26 to 66 [mean = 45.55, standard deviation (SD) = 6.43] years. About half (49.2% and 50.8%, respectively) of the patients were from urban and rural areas. Most (94.1%) patients were married, 4.1% were divorced, and 1.8% were widowed. They had received a mean of 10.18 (SD = 3.32) years of education. Most (77.0%) patients were employed, 17.9% were housewives, and 5.1% were retired. The time since diagnosis for patients ranged from 1 to 4 weeks. All patients were receiving medical treatment at the time of study participation. Thirty-eight percent of the patients had just undergone mastectomy and still been receiving postoperative anti-inflammatory therapies, 13.1% had been undergoing chemotherapy, 48.9% had been undergoing chemotherapy after mastectomy.

#### Healthy control sample

We recruited healthy women for the control group using data from medical examination centers in Changsha and the surrounding area. A letter explaining the study procedure was sent to eligible women, who were then contacted by telephone to arrange face-to-face interviews if they were interested in participating. Eligible women had self-reported good physical health and spoke Chinese. Women with the following conditions were excluded: (1) history of any type of cancer, (2) known untreated or unstable major medical condition, (3) known major psychiatric or neurological disorder that would interfere with completion of the measures, and (4) history of substance abuse.

**Figure 2:**
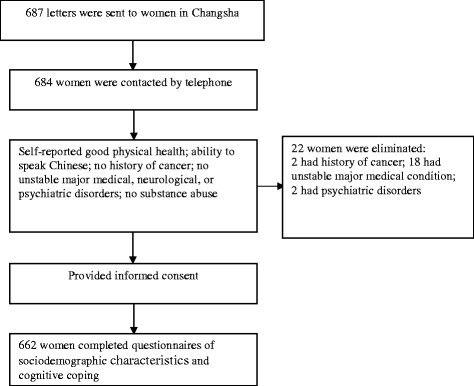
**Process for controls selection.**

Of 687 women to whom letters were sent, 684 were successfully contacted by telephone. Twenty-two of these women were eliminated on the basis of the exclusion criteria; a total of 662 (96.4%) women participated in the study (see Figure 2). Participant age ranged from 30 to 63 (mean = 44.99, SD = 5.63) years. About half (50.5% and 49.5%, respectively) of the women were from urban and rural areas. Most (95.2%) women were married, 2.6% were divorced, and 2.2% were widowed. They had received a mean of 10.30 (SD = 3.63) years of education. Most (77.8%) women were employed, 18.7% were housewives, and 3.5% were retired. No demographic variable differed significantly between patients and healthy control subjects (Table 1).

**Table 1:** **Demographic data of the two samples and medical data of the patients**

	**Patient group**	**Healthy control group**	_***t*or**_ ***χ*** ^**2**^	***p***
	**(n = 665)**	**(n = 662)**		
Years of age (SD)	45.55(6.43)	44.99(5.63)	1.682	0.093
Years of schooling (SD)	10.18(3.32)	10.30(3.63)	−0.621	0.535
Place of residence (%)			0.217	0.641
Urban	49.2	50.8		
Rural	50.5	49.5		
Marital status (%)			2.612	0.271
Married	94.1	95.2		
Widowed	1.8	2.2		
Divorced	4.1	2.6		
Employment status (%)			2.228	0.328
Employed	77.0	77.8		
Housewife	17.9	18.7		
Retired	5.1	3.5		
Stage (%)				
I	14.8			
II	85.2			
Weeks since diagnosis (SD)	0.35(0.84)			
Therapy type (%)				
Mastectomy	38.0			
Chemotherapy	13.1			
Mastectomy with chemotherapy	48.9			

### Data collection

After participants provided informed consent, trained psychology students administered structured questionnaires in face-to-face interviews to collect information on sociodemographic characteristics and cognitive emotion regulation strategies from participants in both groups, and medical characteristics and QOL from patients with breast cancer.

### Measures

The following demographic data were collected: age, years of education, long-term area of residence (urban/rural), marital status, and employment status.

#### Cognitive emotion regulation strategies

The Cognitive Emotion Regulation Questionnaire (CERQ) was the first instrument developed to explicitly measure cognitive strategies for emotion regulation that individuals may use in response to threatening or stressful life events [11]. The 36-item CERQ contains nine conceptually distinct subscales: five for adaptive strategies (acceptance, positive refocusing, refocusing on planning, positive reappraisal, and putting into perspective) and four for maladaptive strategies (self-blame, rumination, catastrophizing, and blaming others). Item responses are structured by a five-point Likert scale ranging from 1 [(almost) never] to 5 [(almost) always]. Subscale scores are obtained by summing component item scores (range, 4–20), with higher scores indicating greater use of a specific cognitive strategy. The CERQ can be used to measure general coping style (trait) or response to a specific event (state). The CERQ and the Chinese version of this instrument (CERQ-C) have shown good reliability and validity [11,24]. The CERQ-C was used in the present study, and the subscales had good internal consistency, with alpha coefficients ranging from 0.65 (putting into perspective) to 0.91 (refocusing on planning).

#### Quality of life

The Functional Assessment of Cancer Therapy for Breast Cancer (FACT-B) scale was used to assess multidimensional QOL in patients with breast cancer [25]. The scale consists of 36 items in five domains: physical well-being (PWB), social/family well-being, emotional well-being, functional well-being (FWB), and breast cancer–specific concerns (BCS). Item responses are structured by a five-point scale (0 = not at all, 1 = little bit, 2 = somewhat, 3 = quite a bit, 4 = very much). Total and subscale scores are calculated by summing item scores. Higher scores indicate better functional status. The internal consistency and content validity of the scale have been demonstrated in a sample of Chinese patients with breast cancer, with Cronbach’s alpha coefficients for the subscales ranging from 0.59 (BCS) to 0.85 (PWB) [26]. The Chinese version of the FACT-B scale was used in the present study, and subscales showed good internal consistency [*α* = 0.53 (BCS) to 0.89 (FWB)].

### Data analyses

G*Power 3 [27] was used for estimation of sample size in this study. Sample size was calculated using an independent-samples *t*-test with the parameters *α* = 5% (two sided) and 1 – *β* = 95%. According to the results of our preliminary study, we selected a small effect size (*d* = 0.2) [28]. These calculations indicated that 651 participants per group were needed. To account for possible dropouts, we increased this value by about 5% to 684 participants per group.

Descriptive analyses, *t*-tests, logistic regression analyses, and multiple regression analyses were performed using SPSS 18.0 software [29]. Independent-samples *t*-tests were performed to examine differences in CERQ-C subscale scores between patients and healthy control subjects. Logistic regression analysis was then performed, with group assignment serving as the binary dependent variable and the nine cognitive strategies serving as independent variables, to identify strategies that best distinguished the two groups. Pearson correlations were performed to examine the relationships between the nine cognitive emotion-regulation strategies and QOL in patients. Multiple regression analyses were performed to examine the effects of cognitive coping strategies on QOL in patients after controlling for sociodemographic and medical variables.

## Results

### Differences in cognitive emotion regulation strategies between groups

Means of reported use of the nine cognitive emotion-regulation strategies were calculated for both groups. Significant differences between groups were found for eight of the nine cognitive strategies (*p* < 0.01; Cohen’s *d* = 0.16–0.69; Table 2). Compared with control subjects, patients reported significantly less frequent use of self-blame, rumination, positive refocusing, refocus on planning, positive reappraisal, and blaming others, and significantly more frequent use of acceptance and catastrophizing. Use of the putting into perspective strategy did not differ between groups.

**Table 2:** **Differences in the reporting of cognitive emotion regulation strategies between patient and healthy control sample**

	**Patient group**	**Healthy control group**	***t***	***p***	**|Cohen’s d|**
	**(** ***n*** **= 665)**	**(** ***n*** **= 662)**			
Self-blame	11.24(3.55)	11.72(2.34)	−2.889	0.004	0.16
Acceptance	13.65(3.03)	13.22(2.40)	2.912	0.004	0.16
Rumination	10.25(3.41)	11.50(2.84)	−7.259	<0.001	0.40
Positive refocusing	10.70(3.36)	12.48(2.77)	−10.513	<0.001	0.58
Refocus on planning	13.68(3.11)	14.65(3.25)	−5.573	<0.001	0.30
Positive reappraisal	11.85(3.25)	14.02(3.00)	−12.604	<0.001	0.69
Putting in perspective	10.11(2.30)	10.11(2.57)	−0.060	0.952	—
Catastrophizing	10.48(3.45)	8.52(3.00)	11.030	<0.001	0.61
Blaming others	9.56(3.00)	10.17(3.00)	−3.561	<0.001	0.20

### Prediction of group membership: logistic regression analysis

Inclusion of the nine cognitive emotion-regulation strategies as independent variables in logistic regression analysis yielded significant model results (*χ*^2^_(9)_ = 495.856, *p* < 0.001) explaining 31.2% of the variance (Cox and Snell *R*^2^). This model enabled correct classification of control subjects and patients in 78.4% of cases. The Wald statistic was used to determine the significance of the contributions of independent variables. The logistic regression coefficient (B) was used to determine the relative influence of separate independent variables. All nine strategies contributed significantly and independently to the prediction of group membership (Table 3). Catastrophizing was the best predictor (B = −0.35), followed by acceptance (B = −0.29), showing that patient group membership was associated with greater reported use of these strategies. The third most significant predictor was positive reappraisal (B = 0.23), and patient group membership was related to less reported use of this strategy.

**Table 3:** **Cognitive emotion regulation strategies distinguishing patient and control sample membership: logistic regression analysis (** ***n*** **= 1327)**

	**B**	**SEB**	**Wald**	***p***
Self-blame	0.08	0.03	9.19	0.002
Acceptance	−0.29	0.03	91.35	<0.001
Rumination	0.20	0.03	59.90	<0.001
Positive refocusing	0.14	0.03	26.40	<0.001
Refocus on planning	−0.07	0.03	5.46	0.019
Positive reappraisal	0.23	0.03	59.20	<0.001
Putting in perspective	0.10	0.03	7.66	0.006
Catastrophizing	−0.35	0.03	128.61	<0.001
Blaming others	0.16	0.02	41.21	0.003

Total explained variance (Cox and Snell R^2^): 31.2%.

Significance model: *χ*2 _(9)_ = 495.856, *P* < 0.001

### Relationships between cognitive emotion regulation strategies and quality of life in patients

Out of 665 patients, 621 completed completed the FACT-B scale. All CERQ-C subscale scores were significantly correlated with FACT-B total scores, with Pearson correlation coefficients ranging from 0.17 (blaming others) to 0.57 (acceptance; Table 4). These correlations were negative in five cases (self-blame, rumination, putting into perspective, catastrophizing, and blaming others) and positive in four cases (acceptance, positive refocusing, refocusing on planning, and positive reappraisal).

**Table 4:** **FACT-B subscales: descriptive, Pearson correlations with CERQ subscales (** ***n*** **= 621)**

**CERQ**	**FACT-B**					
	**PWB**	**SFWB**	**EWB**	**FWB**	**BCS**	**Total**
	**M(SD)**					
	**16.89(5.05)**	**19.94(5.13)**	**13.50(5.23)**	**8.58(6.26)**	**25.88(3.96)**	**84.80(18.98)**
Self-blame	−0.24**	−0.22**	−0.24**	−0.22**	−0.12**	−0.28**
Acceptance	0.48**	0.14**	0.55**	0.55**	0.32**	0.57**
Rumination	−0.24**	−0.24**	−0.41**	−0.33**	−0.19**	−0.39**
Positive refocusing	0.32**	0.20**	0.31**	0.30**	0.10**	0.34**
Refocus on planning	0.34**	0.28**	0.28**	0.38**	0.15**	0.40**
Positive reappraisal	0.33**	0.31**	0.36**	0.47**	0.16**	0.46**
Putting in perspective	−0.31**	−0.01	−0.34**	−0.32**	−0.20**	−0.32**
Catastrophizing	−0.36**	−0.18**	−0.63**	−0.54**	−0.22**	−0.54**
Blaming others	−0.22**	−0.06	−0.08*	−0.15**	−0.12**	−0.17**

Note: *p < 0.05, **p < 0.01.

### Effects of cognitive emotion regulation strategies on quality of life in patients

The results of multiple regression analyses are presented in Table 5. In step 1, sociodemographic variables significantly predicted QOL (*F* = 9.849, *p* < 0.001); they accounted for 7.4% of the variance in QOL, but only the regression coefficients of area of residence and marital status were significant. In step 2, medical variables significantly predicted QOL after controlling for sociodemographic variables (*F* = 10.197, *p* < 0.001), the *R*^2^ change was 0.026, implying that the medical variables together could account for 2.6% of the variance in QOL, but the regression coefficient was significant only for therapy type. In step 3, cognitive emotion-regulation strategies predicted QOL after controlling for sociodemographic and medical variables (*F* = 37.627, *p* < 0.001); the *R*^2^ change was 0.407, implying that the nine cognitive emotion-regulation strategies together could account for 40.7% of the variance in QOL, but the regression coefficients of four strategies (positive refocusing, refocusing on planning, putting into perspective, and blaming others) were not significant.

**Table 5:** **Effect of CERQ subscales on QOL in patients: Multiple Regression Analyses (Method = enter;** ***n*** **= 621)**

	**B**	**SEB**	***t***	***F***	**R** ^**2**^	**Adjusted R** ^**2**^	**R** ^**2**^ **change**
Step 1				9.849***	0.074	0.067	0.074
Age	0.05	0.02	0.43				
Years of schooling	−0.24	−0.04	−0.87				
Place of residence	−10.03	−0.26	−5.29***				
Marital status	−7.09	−0.15	−3.91***				
Employment status	−2.34	−0.07	−1.63				
Step 2				10.197***	0.100	0.098	0.026
Stage	−1.79	−0.04	−0.66				
Weeks since diagnosis	2.50	0.10	1.82				
Therapy type	−2.91	−0.12	−2.16*				
Step 3				37.627***	0.507	0.483	0.407
Self-blame	−0.42	−0.09	−2.38*				
Acceptance	2.52	0.43	7.51***				
Rumination	−0.81	−0.14	−2.55**				
Positive refocusing	0.11	0.02	0.34				
Refocus on planning	0.10	0.02	0.26				
Positive reappraisal	0.53	0.10	2.82**				
Putting in perspective	−0.79	−0.09	−1.67				
Catastrophizing	−1.73	−0.33	−3.97**				
Blaming others	−0.37	−0.06	−1.39				

Note: * *p* < 0.05, ** *p* < 0.01, *** *p* < 0.001.

## Discussion

To our knowledge, the present study is the first to report on the use of cognitive emotion regulation strategies and the effects of these strategies on QOL among Chinese women undergoing breast cancer treatment.

Consistent with our first hypothesis, the results showed that cognitive emotion regulation strategies differed significantly between women with breast cancer and physically healthy women. Compared with healthy women, women newly diagnosed with breast cancer reported more frequent use of catastrophizing, a maladaptive cognitive emotion regulation strategy, and less frequent use of adaptive strategies (positive refocusing, refocusing on planning, and positive reappraisal). These findings are similar to those of previous research in patient and general-population samples [15,22]. However, it is still unknown to us that whether those differences existed before the diagnosis or just were patients’ reflection of the stress induced by the disease. In either case, emotion regulation strategies do play a crucial role when an individual encounters negative events and stress. Catastrophizing involves exaggerated threat appraisal and thoughts that explicitly emphasize the terror of an experience. In general, a catastrophizing coping style is positively related to depression and anxiety [11]. Refocusing on planning refers to thinking about what steps to take and how to handle a negative event. Carver et al. [30] showed that the use of planning as a coping strategy was negatively related to anxiety. Min et al. [31] reported that refocusing on planning was the common strategy contributing to resilience and depression. Positive refocusing, characterized by thinking about joyful and pleasant matters instead of a negative event, is negatively related to depression [11]. Positive reappraisal refers to the attachment of a positive meaning to a negative event in the context of personal growth. Garnefski et al. [11] and Carver and Scheier [30] showed that this strategy is negatively related to anxiety. Taken together, these findings imply that women who were recently informed the diagnosis of breast cancer have many fears related to the disease, they may not yet have had a chance to process all the information and events surrounding their diagnosis and let alone to find positive aspects of their condition.

In contrast to previous findings [22,23], women newly diagnosed with breast cancer more often reported acceptance and less often reported self-blame, rumination, and blaming others than did physically healthy women. Self-blame refers to thoughts of blaming oneself for what one has experienced. Rumination refers to thinking about the feelings and thoughts associated with a negative event. Among maladaptive strategies, these strategies have shown the strongest correlations with depression [11,32,33]. The infrequency of rumination suggests that women newly diagnosed with breast cancer avoid cognition related to the disease. Acceptance refers to thoughts of accepting what one has experienced and resigning oneself to what has happened. Blaming others refers to thoughts of putting the blame for what one has experienced on others. Blaming someone else, an external attribution style, has been associated with poorer emotional well-being [34]. Patients’ greater use of acceptance and less-frequent blaming of others are partly consistent with the characteristics of a type C coping style [35], which describes individuals as being “cooperative and appeasing, unassertive, patient, inexpressive of negative emotions and compliant with external authorities.” Thus, cognitive coping is to some extent consistent with behavioral coping in women with breast cancer.

The findings that greater use of catastrophizing and acceptance, and less-frequent use of positive reappraisal, strongly and independently predicted patient group membership agree in part with previously reported findings [36] that clinical individuals with symptoms of depression and anxiety commonly practiced more catastrophizing and less positive reappraisal than controls, leading to poor adjustment to stress. The less-frequent use of rumination and blaming others may reflect cognitive coping strategies consistent with the type C coping style. As previous studies have established a possible link between Type C personality and cancer [35,37], women with other cancer may have similar cognitive coping pattern; however, individuals with other illnesses, such as cardiovascular disease, may present different patterns of cognitive emotion regulation (e.g., Type A behavior pattern [37]).

Previous studies have confirmed that the effects of stress on health outcomes depend on how a person copes with stress [38]. In this study, cognitive coping was correlated with overall QOL and all QOL domains. Patients reporting more frequent use of maladaptive strategies (self-blame, rumination, catastrophizing, and blaming others) had worse perceived QOL, whereas those reporting more frequent use of adaptive strategies (acceptance, positive refocusing, refocusing on planning, and positive reappraisal) had better perceived QOL. The strategies of catastrophizing, acceptance, and positive reappraisal were strongly related to QOL, consistent with the findings of previous studies. For example, Jacobsen et al. [18] and Khan et al. [19] found that catastrophizing was related to fatigue and pain in women with breast cancer. Acceptance has been found to be beneficial for both psychological adjustment and QOL in patients with breast cancer [13,19]. Positive reappraisal has been shown to be a good predictor of positive mood, perceived health, and posttraumatic growth in women with breast cancer [39]. Surprisingly, however, patients in this study reporting more frequent use of the putting into perspective adaptive strategy had worse perceived QOL. This strategy is arguably similar to the concept of social comparison [11], and more frequent use of it may involve more attention to information on breast cancer treatment and similar or worse related events, which may reduce QOL.

Multiple regression analyses revealed that sociodemographic and medical factors had significant effects on QOL. Patients in relationships and those from urban areas had better perceived QOL than did patients who were divorced or widowed and those from rural areas, respectively; patients undergoing chemotherapy had worse perceived QOL than did those undergoing surgery alone. These findings are consistent with the results of previous studies [40,41]. Disease stage and time since diagnosis had no significant effect on QOL, however it is important to take into consideration that all patients were at early stage of the disease and enrolled in this study shortly after diagnosis (within a month). After controlling for sociodemographic and medical variables, cognitive coping had a significant effect on QOL. Maladaptive strategies (self-blame, rumination, and catastrophizing) had negative effects and adaptive strategies (acceptance and positive reappraisal) had positive effects on QOL. These findings are consistent with previous research demonstrating that cognitive coping mediates and moderates associations between various stressors and psychosomatic adjustment [14,42]. Clinical staff should pay particular attention to catastrophizing, which showed the largest difference among maladaptive strategies between the patient and control samples, to reduce patients’ fear of the disease. Clinical education about breast cancer should be implemented as early as possible. Acceptance had a positive effect on QOL. Some authors have argued that this strategy has distinct effects as an active process of self-affirmation and as a passive form of resignation to negative experiences [16]. In the current study, acceptance results reflected self-affirmation, typically considered to be a functional coping response, as accepting the reality of a situation implies a certain attempt to deal with that situation. The results thus imply that cognitive acceptance was more important than behavioral acceptance among our patients. Folkman et al. [38] argued that the value of positive reappraisal was not limited to the alleviation of distress, as positive interpretation of a stressful event should lead individuals to continue active and effective coping actions. In this study, positive reappraisal showed the largest difference among adaptive strategies between the patient and control samples. Thus, future research should focus on identifying and developing professional interventions that improve patients’ ability to accept stressors as real during primary appraisal and to attach positive meanings to stressful events through positive reappraisal.

The limitations of the present study include its cross-sectional nature, which prevented us from drawing conclusions about the development, course, and changes in QOL and patterns of cognitive emotion regulation over time. Thus, longitudinal studies should be conducted to address the potential bidirectional relationship between reported use of coping strategies and the experience of illness. Also, the assessment of cognitive emotion regulation strategies was based on a self-reported measure, which may have introduced bias (e.g., social desirability) and in future studies, the inclusion of other assessments may be useful to validate these findings. Thirdly, as the data of patients uninformed with the disease were not available, findings in this study could not be generalized to all women with breast cancer.

## Conclusions

Despite its limitations, the findings of the present study provide preliminary evidence for a specific cognitive emotion regulation style among women newly diagnosed with breast cancer, characterized by more frequent use of catastrophizing and acceptance and less frequent use of positive reappraisal, self-blame, rumination, positive refocusing, refocusing on planning, and blaming others. The present study also provides empirical evidence that this cognitive emotion regulation style is associated with QOL in women undergoing treatment for breast cancer: catastrophizing, rumination, and self-blame had negative effects and acceptance and positive reappraisal had positive effect on QOL in these patients. The results of this study highlight the importance of including the assessment of cognitive emotion regulation strategy use in making interventions aiming to improve QOL in women with breast cancer.

## Abbreviations

QOL: Quality of life
CERQ: Cognitive Emotion Regulation Questionnaire
CERQ-C: Chinese version of the Cognitive Emotion Regulation Questionnaire
FACT-B: Functional Assessment of Cancer Therapy for Breast Cancer
PWB: Physical well-being
SFWB: Social/family well-being
EWB: Emotional well-being
FWB: Functional well-being
BCS: Additional concerns on breast cancer
